# The effect of single versus group culture on cumulus-oocyte complexes from early antral follicles

**DOI:** 10.1007/s10815-025-03404-w

**Published:** 2025-01-28

**Authors:** Mohammadreza Ebrahimi, Laura Mara, Sara Succu, Sergio Domenico Gadau, Maria Grazia Palmerini, Fabrizio Chessa, Maria Dattena, Francesca D. Sotgiu, Valeria Pasciu, Ilaria Antenisca Mascitti, Guido Macchiarelli, Alberto Maria Luciano, Fiammetta Berlinguer

**Affiliations:** 1 Department of Veterinary Medicine, University of Sassari, Via Vienna 2, Sassari, Italy; 2 Department of Animal Science, Agricultural Research Agency of Sardinia, 07100 Sassari, Italy; 3 Department of Life, Health and Environmental Sciences, University of L’Aquila, 67100 L’Aquila, Italy; 4 Reproductive and Developmental Biology Laboratory (ReDBioLab), Department of Veterinary Medicine and Animal Sciences, University of Milan, Via dell’Università, 6-26900 Lodi, Italy

**Keywords:** Long in vitro culture (LIVC), Group culture, Assisted reproductive technology, Cumulus-oocyte complexes (COCs), Early antral follicle, In vitro growth

## Abstract

**Purpose:**

This study aimed to evaluate the effectiveness of single versus group culture strategies for cumulus-oocyte complexes (COCs) derived from early antral follicles (EAFs), with the goal of optimizing culture conditions to increase oocyte availability for assisted reproductive technologies.

**Methods:**

COCs isolated from EAFs (350–450 µm) from sheep ovaries were cultured in TCM199 medium supplemented with 0.15 µg/mL Zn^++^ as zinc sulfate, 10^−4^ IU/mL FSH, 10 ng/mL estradiol, 50 ng/mL testosterone, 50 ng/mL progesterone, and 5 µM Cilostamide. After 5 days of long in vitro culture (LIVC), COCs underwent in vitro maturation. This study investigated the effects of single and group culture conditions on COCs, focusing on morphology (integrity of oocyte-granulosa cell complex), viability, oocyte diameter, chromatin configuration, and ultrastructure. Additional factors influencing developmental competence were assessed, including global transcriptional activity, gap junction communication, and meiotic competence. Intracellular reactive oxygen species (ROS) levels and mitochondrial activity were also measured.

**Results:**

No significant differences were observed between groups in terms of morphology, viability, oocyte diameter, chromatin configuration, ROS levels, or mitochondrial activity. However, group culture resulted in ultrastructural changes, with a notable reduction in global transcriptional activity, an increase in active gap junctions, and a higher rate of meiosis resumption (*p* < 0.01).

**Conclusion:**

Overall, group culture of COCs derived from sheep EAFs promoted meiosis resumption, suggesting that this approach could improve in vitro culture techniques, increase the availability of mature gametes, and support fertility preservation programs.

**Graphical abstract:**

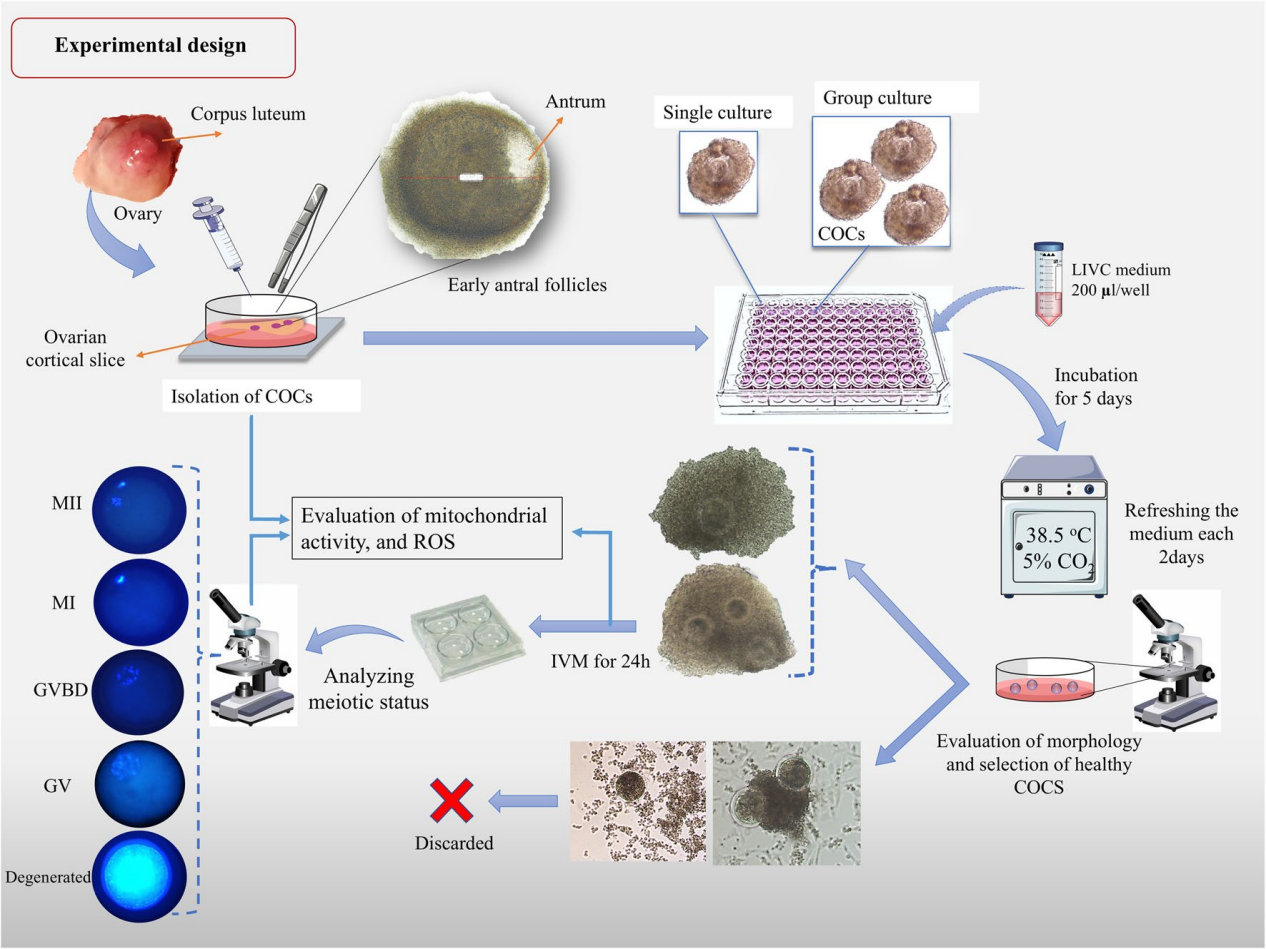

**Supplementary Information:**

The online version contains supplementary material available at 10.1007/s10815-025-03404-w.

## Introduction

Throughout the lifespan of female mammals, only a small proportion of follicles complete their growth and reach ovulation, while most undergo atresia at different developmental stages [1]. Therefore, given the abundance of immature follicles in the ovary, leveraging these follicles could significantly expand fertility preservation options for valuable genetic animals and cancer patients. Although assisted reproductive technologies (ARTs) can successfully produce mature oocytes and embryos from cumulus-oocyte complexes (COCs) collected from fully grown antral follicles, they face challenges in promoting the growth and maturation of earlier-stage ovarian follicles that have not yet reached their final developmental stage [2]. Accordingly, extensive research has been conducted to develop a method for enhancing the utilization of the ovarian reserve and preserving genetic inheritance by leveraging non-ovulatory follicles, which are typically untapped in conventional in vitro embryo production procedures [3–5]. Among the untapped follicles, early antral follicles (EAFs) are promising options for fertility preservation due to their greater abundance compared to antral follicles and shorter growth phase duration than secondary follicles [3, 6, 7]. Accordingly, innovative culture systems have been developed to enhance the production of competent oocytes from EAFs in various animal species, including sheep [3], goats [8], humans [9], and cows [6]. However, the low yield of metaphase II (MII) oocytes and embryos underscores the challenges of replicating natural physiological conditions to support optimal oocyte development [6, 8, 10, 11]. This limitation likely arises from the isolation of COCs from their natural follicular environment and accompanying cells, resulting in premature meiotic resumption [12] and insufficient protein and RNA synthesis [13–15]. To address this, phosphodiesterase type 3 (PDE3) inhibitors are proposed to maintain cAMP levels and support oocyte-cumulus cell communication [16–18]. However, despite these advances, insufficient protein and RNA synthesis remains an inherent challenge, representing one of the primary barriers to achieving optimal in vitro development of oocytes derived from pre-antral follicles. This shortfall highlights the need for more advanced in vitro culture systems that closely replicate the native follicular environment. One approach involves simulating the intraovarian microenvironment through group culture [19] or co-culture with cumulus or granulosa cells [20, 21]. It is well-documented across species that granulosa cells play a pivotal role in the developmental competence of oocytes by providing essential nutrients and regulatory signals [19–24]. Therefore, when COCs are isolated from the follicles, they retain significantly fewer attached granulosa cells than intact follicles—approximately 8000 versus 1,390,000 in pigs [21]. Moreover, it has been documented that growing follicles in the mouse ovary produce signals that promote the local growth of other follicles [23]. This observation suggests a potential, though unconfirmed, similar mechanism in polyovulatory species such as sheep. These findings imply that successful in vitro culture may need additional factors beyond standard media additives to more accurately replicate the intraovarian signaling environment.

Accordingly, to mimic the intraovarian microenvironment and achieve fully developed oocytes in vitro, this study employs a long in vitro culture (LIVC) system. This approach, successfully employed in cows [25, 26] and sheep [3, 27], involves isolating and 3D culturing COCs from EAFs. This system enables efficient bidirectional communication between the oocyte and granulosa cells, supporting oocyte growth while preventing premature meiotic resumption. This is achieved by stimulating cAMP production with a physiological level of FSH and inhibiting cAMP hydrolysis with Cilostamide, an oocyte-specific PDE3 inhibitor [28].

This study aims to evaluate whether a group culture system, compared to a single culture system, more effectively supports the development and growth of COCs collected from sheep EAFs, thereby enhancing the yield of mature oocytes. Sheep models offer valuable insights due to their physiological similarities to humans, making them an excellent choice for studying reproductive physiology and advancing ARTs with potential applications in human medicine [29]. Additionally, this approach could contribute to preserving valuable and endangered animals with seasonal reproduction patterns and enhance our ability to develop a reliable follicle culture system.

## Material and method

All chemicals and reagents used in this study were purchased from Sigma Chemicals Company, unless otherwise specified.

### Experimental design

The experimental design consisted of isolating COCs (GV stage) from EAFs and subjecting them to the long in vitro culture process (LIVC) for 5 days, either individually or in groups. The 5-day duration was chosen based on previous studies showing that this is the minimum time required for oocytes within EAFs to reach meiotic competence [3]. A comprehensive series of assessments were conducted both before and after the LIVC procedure, encompassing various parameters such as COC morphology, cumulus cell viability, ultrastructural evaluations, gap junction communication, oocyte diameter, global transcriptional activity, chromatin configuration, levels of reactive oxygen species (ROS), and mitochondrial activity and distribution patterns. Following these assessments, to investigate the potential of meiosis resumption, the in vitro-grown COCs were subjected to an in vitro maturation (IVM) process (Supplementary Fig. 1).

### Ovary collection and isolation of cumulus-oocyte complexes

Sheep ovaries (Sarda sheep) were collected during the non-breeding season from a local slaughterhouse and transported to the laboratory within 1 h. To ensure ideal conditions during transportation, they were placed in a 50-mL tube filled with a 0.9% saline solution and then in a thermos flask maintained at 37 °C. Upon arrival at the laboratory, the ovaries were rinsed with a holding medium (H-TCM199) consisting of TCM199 supplemented with 20 mM HEPES, 1790 IU/L Heparin, 0.4% BSA (BSA, fraction V), 0.062 g/L Penicillin G, and 0.010 g/L Streptomycin. All subsequent procedures were performed at a controlled temperature of 38.5 °C using a warm plate. To isolate growing COCs, ovarian cortical slices (1–2 mm thick) were carefully sectioned using a surgical blade and placed in a 6-cm glass Petri dish containing H-TCM199 supplemented with 5 µM Cilostamide (HTCM199-Cilo) [6]. Cilostamide, a specific PDE3A inhibitor, prevents cyclic cAMP hydrolysis. The prevention of cAMP drop has been shown to maintain gap junction functionality between oocytes and cumulus cells [16, 17]. The samples were then examined under a stereomicroscope, and COCs were retrieved by gently puncturing the follicle walls of EAFs (outer diameter, 350–450 µm), using a 21-gauge needle [6]. The isolated COCs were then washed in HTCM199-Cilo and transferred to a pre-warmed Petri dish (38.5 °C with 5% CO_2_) containing base medium (B-TCM199), which consisted of TCM199 supplemented with 2 mM GlutaMAX™ (ThermoFisher Scientific, Milano, Italy), 0.4% fatty acid-free BSA, 0.2 mM sodium pyruvate, 25 mM sodium bicarbonate, 0.1 mM cysteamine, 75 µg/mL kanamycin, 4% polyvinylpyrrolidone (PVP; 360 k molecular weight), 21.3 µg/mL of phenol red, and 5 µM Cilostamide [6]. Each isolation session lasted a maximum of 30 min, yielding 20–30 COCs per session (4–8 COCs per ovary).

### Incorporation of COCs in the LIVC system

Before initiating the LIVC process, the diameter of the oocytes was measured using an inverted microscope (Nikon Diaphot-TMD) equipped with a digital camera (Leica-DFC450) and a heated stage. Only oocytes with a diameter of 110 ± 5 µm (excluding the zona pellucida) that displayed a uniform cytoplasm and were surrounded by five or more complete layers of cumulus cells without any signs of expansion were selected [6].

The selected COCs were then washed in pre-warmed B-TCM199 (38.5 °C, 5% CO₂) and cultured in a 96-well plate (BioCoat™ Collagen I, Becton Dickinson Italia, Milan, Italy), either individually or in groups of three per well. Each well contained 200 µL of B-TCM199 supplemented with 0.15 µg/mL Zn sulfate, 10^−4^ IU/mL FSH (r-hFSH, Gonal-F, Merck-Serono, Roma, Italia), 10 ng/mL estradiol, 50 ng/mL testosterone, and 50 ng/mL progesterone [6]. The plates were incubated in a humidified atmosphere at 38.5 °C with 5% CO₂ for 5 days.

To maintain a stable culture environment, sterile embryo culture tested water was added around the edges of the culture plates. Half of the culture medium volume was replaced with freshly prepared medium on the second and fourth days of culture. Following the culture, only COCs with compact granulosa cells and no morphological indications of degeneration, such as darkened or deformed oocytes, were selected for further analysis or in vitro maturation.

### Evaluation of COCs before and after LIVC

#### Morphology and viability of COCs

The morphology of COCs before (*n* = 96) and after single (*n* = 45) and group (*n* = 70) culture was evaluated using an inverted microscope (Nikon Diaphot-TMD) equipped with a digital camera (Leica DFC450) and a warm plate (38.5 °C). COCs were classified into three grades based on cumulus cell integrity: grade 1, with compact cumulus cell layers, showing no signs of cell expansion or degeneration; grade 2, with multiple cumulus cell layers, lacking cumulus expansion and exhibiting partial disaggregation in the outer cell layers; and grade 3, with significant cumulus cell loss (over 50% of the oocyte surface) and signs of cell degeneration [6, 26].

The viability of COCs before (*n* = 96) and after single (*n* = 48) and group culture (*n* = 70) was assessed using propidium iodide (PI) staining, as previously described [30]. Briefly, COCs were incubated in a culture medium containing 10 µg/mL PI for 5 min (38.5 °C, 5% CO_2_). Then, COCs were rinsed three times in H-TCM119 medium to remove excess PI and examined under an inverted fluorescence microscope (Nikon Diaphot; Nikon) equipped with a standard filter set (excitation, 535 nm; emission, 617 nm). COCs were classified according to the proportion of the oocyte surface covered by cumulus cells stained with PI. Based on the extent of PI staining of cumulus cells, COCs were categorized into three classes: class 1, with less than 25% of the cumulus cells stained; class 2, with 25–50% of cumulus cells stained; and class 3, with more than 50% of cumulus cells stained [31, 32].

#### Ultrastructural analysis

For an in-depth investigation of cytoplasmic maturation, freshly isolated COCs from EAFs (*n* = 2), as well as those after LIVC in both singles (*n* = 2) and groups (*n* = 3), were fixed and processed for transmission electron microscopy (TEM) analysis. Fixation was performed in 2.5% glutaraldehyde in PBS solution. After fixation for 2–5 days at 4 °C, COCs were rinsed in PBS, post-fixed with 1% osmium tetroxide (Electron Microscopy Sciences) and rinsed again in PBS. COCs were then singularly embedded in small blocks of 1% agar of about 5 × 5 × 1 mm in size, dehydrated in ascending series of ethanol (Carlo Erba Reagenti, Milan, Italy), immersed in propylene oxide (BDH Italia, Milan, Italy) for solvent substitution and embedded in epoxy resin EMbed-812 (Electron Microscopy Sciences) for 48 h at 60 °C. Ultrathin Sects. (60–80 nm) were cut with a diamond knife, mounted on copper grids, and contrasted with Uranyless (Uranyl acetate alternative) (TAAB Laboratories Equipment Ltd., Aldermaston, UK) and lead citrate (Electron Microscopy Sciences). They were examined and photographed using a Zeiss EM 10 and a Philips TEM CM100 Electron Microscopes operating at 80 kV. The following parameters were evaluated: type and quality of organelles (e.g., vacuoles and mitochondria), the integrity of the oolemma and zona pellucida (ZP), and the appearance of intercellular contacts, including microvilli and transzonal projections [33].

#### Gap junction communications

To compare the functionality of gap junction communication (GJC), the fluorescent dye Lucifer Yellow (LY) was injected into oocytes before (*n* = 36) and after single (*n* = 23) and group culture (*n* = 26). Briefly, the COCs were transferred into a 50 µL drop of TCM199 supplemented with 0.4% BSA and covered with mineral oil. Subsequently, 8–10 nL (equivalent to 1% of the oocyte volume) of LY (5% w/v in 5 mM lithium chloride) was calculated and injected into the ooplasm using a Narishige microinjection apparatus (Narishige Co., Tokyo, Japan), as previously described [32, 34, 35]. Following a 10-min incubation (38.5 °C, 5% CO₂), dye diffusion into the surrounding cumulus cells was observed using an inverted fluorescence microscope (Nikon Diaphot; Nikon) equipped with a standard fluorescein filter set. Based on the extent of dye spread, COCs were categorized as follows: open, when the dye spread throughout the entire cumulus; partial, when the dye spread to only a limited portion of the cumulus; and closed, when the dye remained confined to the oocyte [32, 36, 37].

#### Oocyte diameter

Oocyte diameter was assessed as an indicator of oocyte quality and competency [38]. Briefly, healthy COCs (Grades 1 and 2) from both single (*n* = 227) and group (*n* = 281) cultures were selected, and their diameters were measured before and after LIVC using an inverted microscope equipped with a digital camera (Leica DFC450), LAS software (Leica Microsystems), and a heated stage (38.5 °C). Diameters were recorded along two perpendicular axes, excluding the zona pellucida.

#### Global transcriptional activity

To further assess the impact of culture conditions on oocyte development, the global transcriptional activity of oocytes was examined as an additional parameter. This analysis was carried out using the Click-iT® RNA Imaging Kit (Invitrogen, Thermo Fisher Scientific), as previously described [39]. Briefly, COCs before (*n* = 46) and after single (*n* = 26) and group culture (*n* = 32) were incubated with 2 mM 5-ethynyl uridine (EU) diluted in LIVC medium for 1 h at 38.5 °C under 5% CO_2_. Cumulus cells were mechanically removed using denuding pipettes. The oocytes were then washed in warm PBS/PVA, fixed in 4% PFA in PBS for 30 min, and washed again in PBS/PVA. Samples were permeabilized with 0.5% Triton-X 100 in PBS for 15 min, briefly washed in PBS/ PVA, and incubated in a Click-iT® reaction cocktail for 30 min. After further washing in Click-iT® reaction rinse buffer in PBS/PVA, oocytes were stained with Hoechst 33,342 for 15 min (10 µg/mL Hoechst 33,342 in PBS/PVA), and, after rinsing in PBS/PVA, mounted on a slide and observed with a Nikon Diaphot fluorescent microscope equipped with a standard fluorescein filter set. Transcriptional activity was classified as strong ( +), moderate (+ / −), or absent ( −) [40].

#### Chromatin configuration

Chromatin configuration was used as a marker of oocyte differentiation and competence acquisition [41]. In brief, the oocytes before (*n* = 100) and after single (*n* = 42) and group (*n* = 48) culture systems were denuded by gentle pipetting and washed with H-TCM199 with 4% BSA. They were then stained with Hoechst dye (5 µg/mL Hoechst 33,342 in H-TCM19 + 0.4% BSA) for 15 min (38.5 °C and 5% CO_2_) under mineral oil. Afterward, the oocytes were rinsed with H-TCM199 + 0.4% BSA and observed under a Nikon Diaphot fluorescent microscope with a standard fluorescein filter set. The oocytes at the GV stage were categorized based on their chromatin configuration (NSN, non-surrounded nucleolus, and SN, surrounded nucleolus), as described before [42].

#### Intracellular level of reactive oxygen species (ROS) and mitochondrial activity and distribution

To assess mitochondrial activity and ROS levels, oocytes were collected at different stages of the culture (before LIVC, after LIVC, and IVM) and underwent a concurrent triple staining procedure as previously described (Succu, et al., 2021). Initially, the oocytes before (*n* = 31) and after single (*n* = 18) and group (*n* = 15) cultures were immersed in phosphate-buffered saline containing 20% Fetal Calf Serum (PBS/20% FCS) and incubated for 30 min at 39 °C with Mito-Tracker Red CM-H2XRos (500 nM; MT-Red, Molecular Probes, Inc., Eugene, OR, USA). Subsequently, after undergoing three washes in PBS/20% FCS, the oocytes were subjected to a 20-min incubation in the same medium with 2′,7′-dichlorodihydrofluorescein diacetate (5 mM; H2DCF-DA, Molecular Probe, Eugene, OR, USA), a molecular probe designed for detecting reactive oxygen species (ROS). After exposure to probes, the oocytes were washed three times in PBS/20%FCS, fixed in 2.5% glutaraldehyde/PBS for at least 15 min, and stored (4 °C) until analysis. The oocytes were stained with Hoechst 33,342 and then mounted on a glass slide for evaluation using confocal microscopy (Leica TCS SP5 CLSM with Leica LAS Lite 170 Image software, Wetzlar, Germany). To assess mitochondrial function, samples were examined with a multiphoton laser to detect MitoTracker Red CM-H2XRos (excitation, 579 nm; emission, 599 nm).

Following IVM, the same procedure was used to analyze cultured COCs in both single (*n* = 13) and group (*n* = 16) cultures. The image analysis software (Leica LAS AF Lite) was used to record the mean fluorescence intensity values for subsequent statistical analysis. Furthermore, the mitochondrial distribution pattern was evaluated before culture (*n* = 34), as well as after single (*n* = 18) and group (*n* = 18) culture, and following IVM (*n* = 13 for single and *n* = 16 for group culture). The patterns were then classified into three distinct groups: “fine,” characterized by homogeneous small granulations dispersed throughout the cytoplasm; “granular,” with homogeneous large granulations distributed throughout the cytoplasm; and “clustered,” marked by particularly heterogeneous large granulations either spread across the entire cytoplasm or located in specific cytoplasmic domains [30].

#### Meiotic competence

The meiotic competence was investigated by subjecting the COCs to standard IVM procedures. Freshly isolated oocytes from antral follicles (*n* = 80) and EAFs (*n* = 43), as well as oocytes from single (*n* = 46) and group (*n* = 42) cultures, were collected and washed in H-TCM199. Subsequently, they were incubated in a 4-well dish (15–20 COCs per well; Nunc, Thermo Scientific, USA) with 450 µL of IVM medium containing TCM-199 supplemented with 4 mg/mL BSA, 100 µM cysteamine, 0.3 mM sodium pyruvate, 1 µg/mL estradiol-17β, 40 µg/mL gentamicin, and 1 IU/mL Pluset (500 IU, p-FSH and p-LH, Serono). The dishes were covered with 200 µL of washed mineral oil, and the COCs were incubated at 38.5 °C in a humidified atmosphere of 5% CO_2_ for 24 h. The meiotic status of the oocytes was evaluated as described in the chromatin configuration assessment section. The oocytes were classified as GV (germinal vesicle), GVBD (germinal vesicle breakdown), MI (metaphase I), or MII (metaphase II).

### Statistical analysis

All experiments were repeated at least three times. Data were analyzed using Minitab® Statistical Software 21.4.1.0 and are expressed as mean ± standard error of the mean (SEM). A Generalized Linear Model was employed to assess the statistical significance of variations observed in oocyte diameter, COCs morphology, viability, changes in GJCs, global transcriptional activity, and chromatin configuration. The active mitochondrial phenotypes were analyzed using the chi-square test. Maturation rate data, fluorescence intensity of mitochondrial activity, and intracellular ROS levels were subjected to one-way ANOVA followed by Tukey’s post hoc test to elucidate differences. Statistical significance was determined for probabilities with values less than 0.05.

## Results

### Effect of LIVC on morphology and viability of COCs

**Fig. 1:**
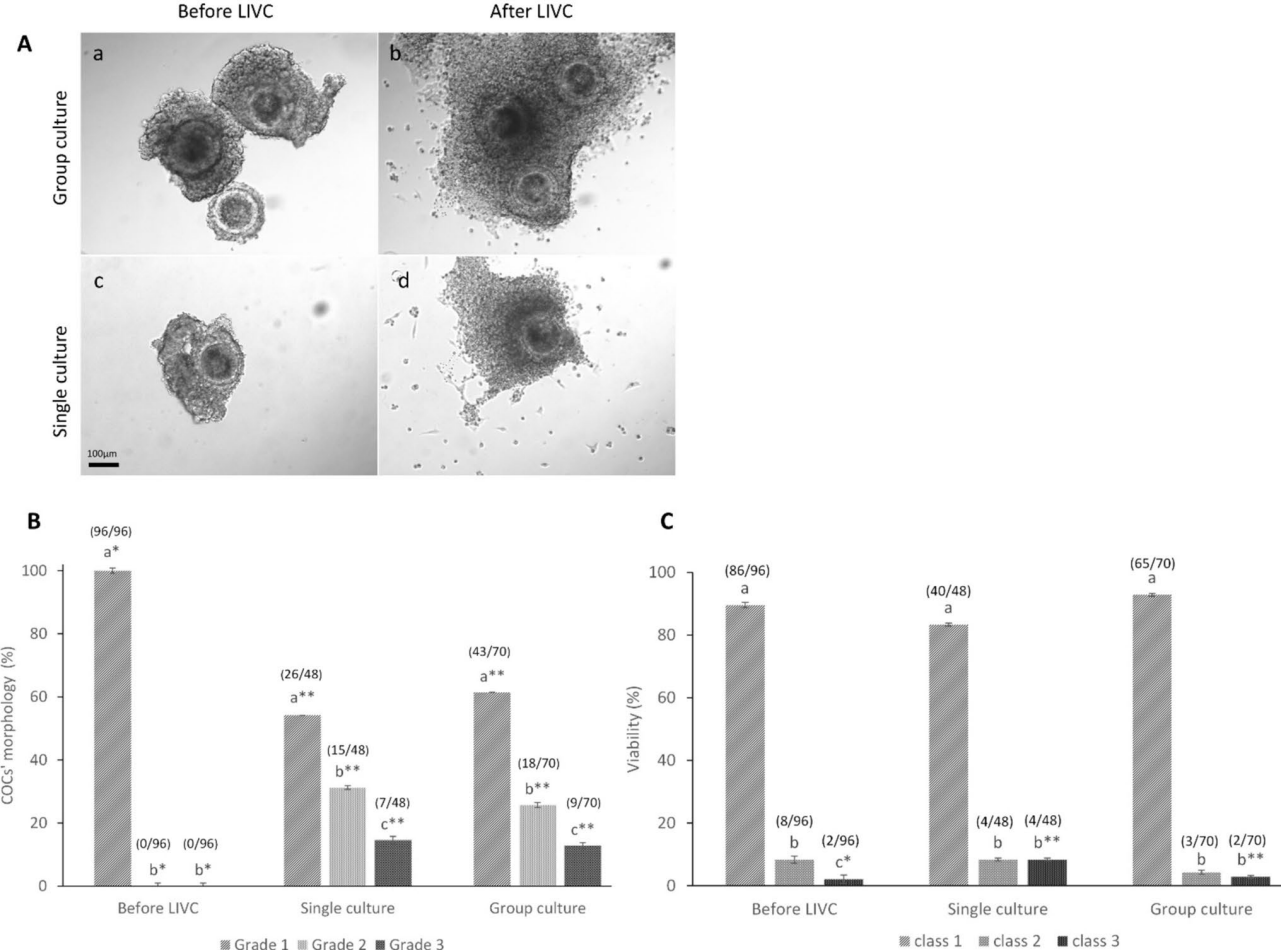
Morphology and viability of COCs in single and group cultures. **A** Representative images displaying the morphology of grade 1 COCs before (a and c) and after 5 days of LIVC in single (d) and group (b) cultures. **B** Graph categorizing COCs into three classes based on morphology: Grade 1 shows COCs with compact cumulus cell layers, exhibiting no expansion or cell degeneration. Grade 2 includes COCs with multiple layers of cumulus cells; these layers lack expansion and feature some disaggregated cells in the outer layer. Grade 3 represents COCs with extensive cumulus cell loss and degeneration. **C** Graph illustrating the percentage of viable COCs, categorized by the percentage of stained cumulus cells into three classes: Class 1 (< 25% stained), Class 2 (25–50% stained), and Class 3 (> 50% stained). Numbers in parentheses indicate the total count of COCs evaluated. Different letters indicate significant differences within the group. Asterisks (*) indicate significant differences between groups (GLM; *p* < 0.05)

COCs were classified into three classes based on the appearance of cumulus cells and the oocyte. Initially, only high-quality COCs with dense granulosa cells were selected (Fig. 1A (a and c)). Therefore, significant alterations in quality were not expected. However, despite this selection, a noticeable decline in the quality of COCs was observed in both experimental groups following LIVC, with no significant differences between them (Fig. 1B). Notably, the group cultured COCs developed tightly packed granulosa cells, forming a cluster-like structure, as illustrated in Fig. 1A(b). Only COCs maintaining structural integrity (Grades 1 and 2) were considered for subsequent analyses. Viability assessments indicated that the percentage of viable COCs (class 1, less than 25% stained cumulus cells) remained stable post-culture, with no significant differences observed between the groups (Fig. 1C). However, the proportion of COCs showing more than 50% stained cumulus cells (class 3) increased significantly post-single culture (*p* < 0.01; Fig. 1C).

### Ultrastructural characteristics

**Fig. 2:**
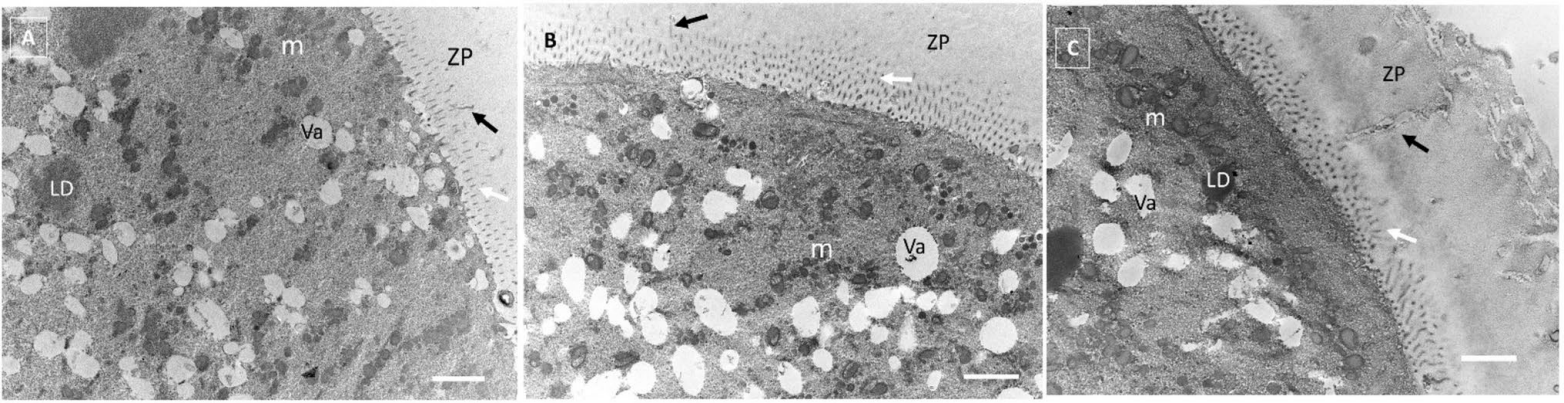
Organelle microtopography of COCs from sheep EAFs before and after single or group culture. Representative TEM micrographs show the ultrastructure of oocytes before (**A**) and after LIVC in both single (**B**) and group (**C**) cultures. The images show the zona pellucida (ZP) crossed by transzonal projections (black arrows) and a perivitelline space containing numerous microvilli (white arrows) protruding from the oolemma. The oolemma features numerous mitochondria (m), electron-negative vacuoles (Va), and electron-dense lipid droplets (LD). Scale bar, 2 µm

Before the LIVC, oocytes were characterized by a thick and continuous ZP with frequent transzonal projections, the absence of a perivitelline space, and the presence of numerous microvilli extending into the ZP. The ooplasm contained irregularly distributed organelles, including electron-pale small vesicles and lipid droplets (Fig. 2A). Mitochondria had a round to ovoid shape and were often found in clusters, either alone or near vesicles or the smooth endoplasmic reticulum. The oolemma displayed numerous small, thin microvilli.

Following the LIVC, oocytes from COCs cultured individually showed a thick and continuous ZP. In contrast, those cultured as a group exhibited a thinner, continuous ZP characterized by an inner electron-pale layer and an outer spongy electron-dense layer. In both groups, a decrease in the number of transzonal projections was observed qualitatively compared to freshly isolated COCs. In the single culture, the ooplasm remained rich in irregularly distributed organelles, including hooded mitochondria. In contrast, the group culture showed a cortical ooplasm rich in electron-pale vacuoles and ovoid or hooded mitochondria, accompanied by fewer vesicles (Fig. 2C). The oolemma in the single culture was folded into numerous irregular microvilli extending into a thin perivitelline space. In contrast, in the group culture, the oolemma had mainly short microvilli protruding into the perivitelline space.

### Gap junction communication (GJCs)

**Fig. 3:**
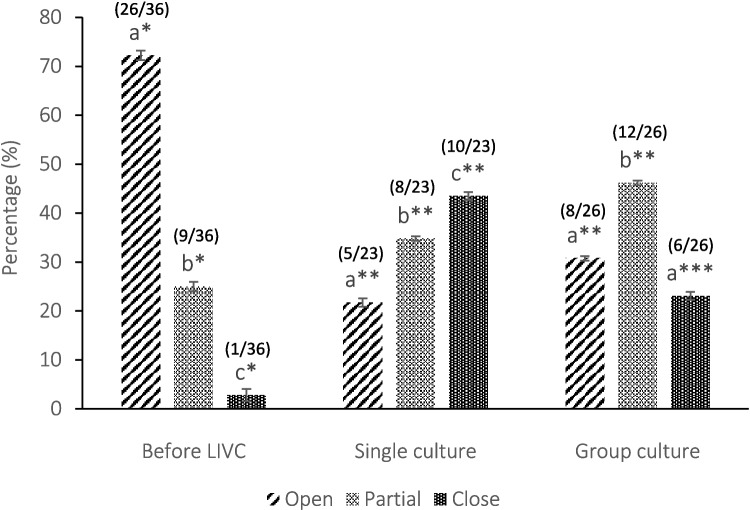
Gap junction communications (GJCs) in COCs before and after LIVC. The bar graph illustrates the distribution of COCs with open, partially open, and closed gap junctions in both single and group cultures, determined through the diffusion of injected Lucifer Yellow dye. Numbers in parentheses indicate the evaluated COCs. Different letters indicate significant differences within the group. Asterisks (*) indicate significant differences between groups (GLM; *p* < 0.05)

Before LIVC, 72.22% and 25% of the oocytes exhibited open or partially open gap junctions, respectively. Following LIVC, a significant decrease in GJCs was observed in both groups (Fig. 3; *p* < 0.000). Notably, most gap junctions in the single culture group were closed (43.47%) or partially closed (34.78%). In contrast, a higher proportion of COCs in the group culture maintained open (30.77%) or partially open gap junctions (46.15%).

### Effect of LIVC on oocyte diameter

**Fig. 4:**
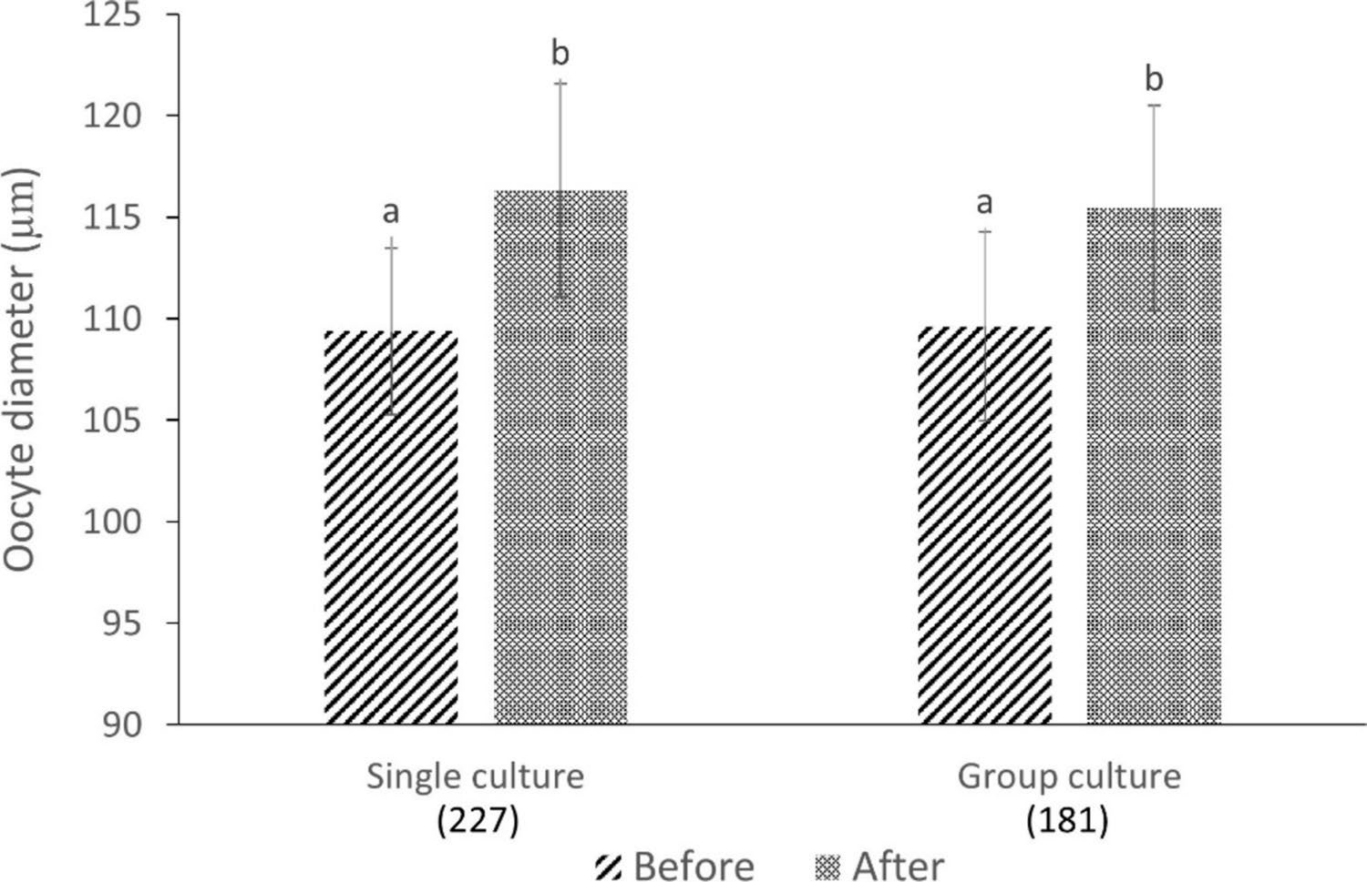
Oocyte diameter. The bar graph depicts the changes in oocyte diameter before and after LIVC in single and group cultures. The numbers in parentheses represent the total count of evaluated COCs in each group. Different letters indicate a significant difference within the same group (GLM; *p* < 0.01). Different letters indicate significant differences within the same group (GLM; *p* < 0.05)

Initially, the mean oocyte diameter was 110.00 ± 5.0 µm in both groups. After 5 days of LIVC, the mean oocyte diameter significantly increased, which reached 116.31 ± 5.2 µm in the single and 115.45 ± 5.0 µm in the group culture. The difference in oocyte growth between the two groups was not statistically significant (Fig. 4, *p* > 0.05).

### Global transcriptional activity

**Fig. 5:**
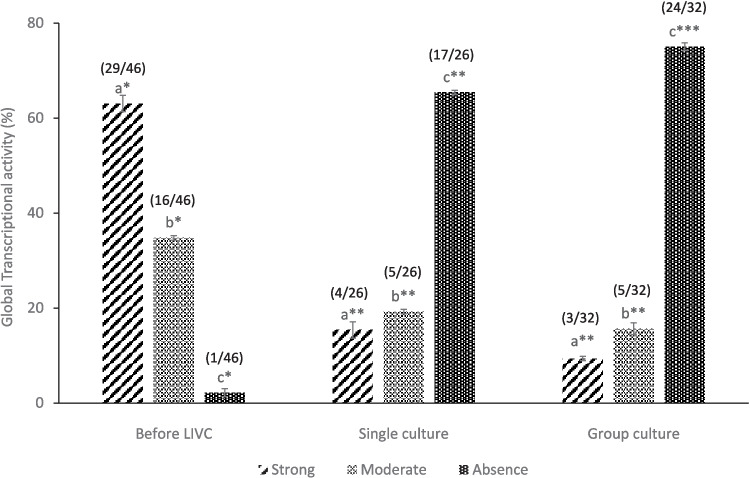
Global transcriptional activity in oocytes before and after LIVC. The bar graph illustrates changes in global transcriptional activity in freshly isolated oocytes from early antral follicles (EAFs) and following LIVC in both single and group cultures. Numbers in parentheses indicate the total count of evaluated COCs. Different letters indicate significant differences within the group. Asterisks (*) indicate significant differences between groups (GLM; *p* < 0.05)

Freshly isolated oocytes were characterized by high transcriptional activity, as depicted in Fig. 5. Following LIVC, most oocytes in both the single and group cultures transitioned into a transcriptional quiescence phase, marked by reduced or absent transcriptional activity. This transition to quiescence was more pronounced in the group culture than in the single culture, indicating a differential impact of the culture conditions on transcriptional activity (Fig. 5, *p* < 0.05).

### Effect of LIVC on chromatin configuration

**Fig. 6:**
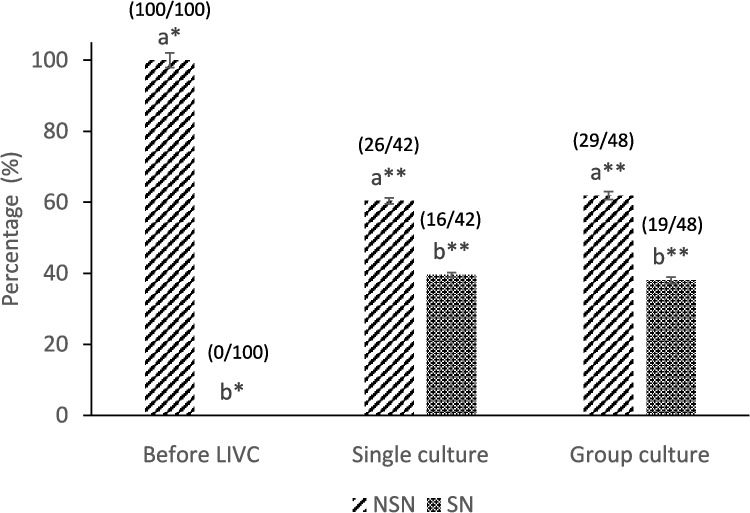
Chromatin configuration before and after LIVC. The bar graph illustrates the transition in chromatin configuration from non-surrounded nucleolus (NSN) to surrounded nucleolus envelope (SN) during LIVC in both single and group cultures. Numbers in parentheses indicate the evaluated COCs. Different letters indicate significant differences within the group. Asterisks (*) indicate significant differences between groups (GLM; *p* < 0.05)

As depicted in Fig. 6, a significant chromatin configuration change was observed during LIVC. All isolated oocytes initially exhibited a non-surrounded nucleolus (NSN) configuration. However, following LIVC, oocytes (38.0% in single and 39.5% in group culture) transitioned to a surrounded nucleolus envelope (SN) configuration, indicative of their acquisition of competence for meiotic resumption. There were no statistically significant differences in the chromatin configurations between the single and group culture conditions (Fig. 6, *p* > 0.05).

### Intracellular level of ROS and mitochondrial activity and distribution

**Fig. 7:**
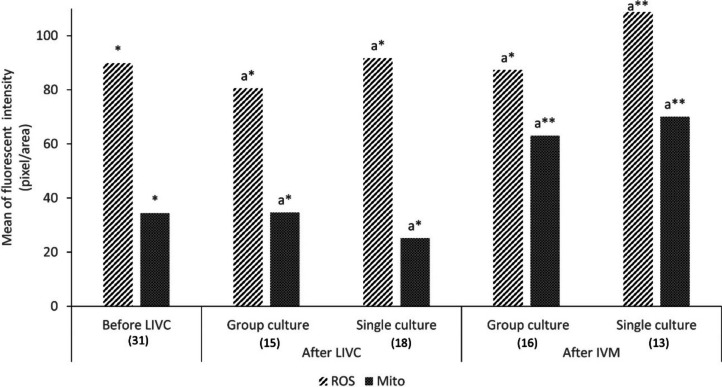
Quantification of mitochondrial and intracellular ROS fluorescence intensity in oocytes before and after LIVC and IVM. This graph illustrates the changes in fluorescence intensity, representing intracellular ROS levels and mitochondrial activity, in oocytes at different stages: before LIVC, after LIVC, and following IVM. Data are shown for both experimental groups. The numbers in parentheses indicate the total count of evaluated COCs. Different letters indicate significant differences within the group. Asterisks (*) indicate significant differences between groups (*p* < 0.05)

Intracellular ROS levels in evaluated oocytes before (*n* = 31) and after single (*n* = 18) or group culture (*n* = 15) remained stable, showing no significant differences (Fig. 7). However, there was a trend toward lower ROS levels in the group culture compared to the individual culture, although this difference did not reach statistical significance (*p* < 0.086). This trend became more pronounced after IVM, with oocytes derived from the single culture (*n* = 13) showing higher ROS levels compared to those from the group culture (*n* = 16) (Fig. 7, *p* < 0.086).

Mitochondrial activity, assessed by measuring fluorescence intensity, showed no statistically significant differences between the experimental groups. Nevertheless, a notable increase in mitochondrial activity was observed following IVM (Fig. 7, *p* < 0.05).

**Fig. 8:**
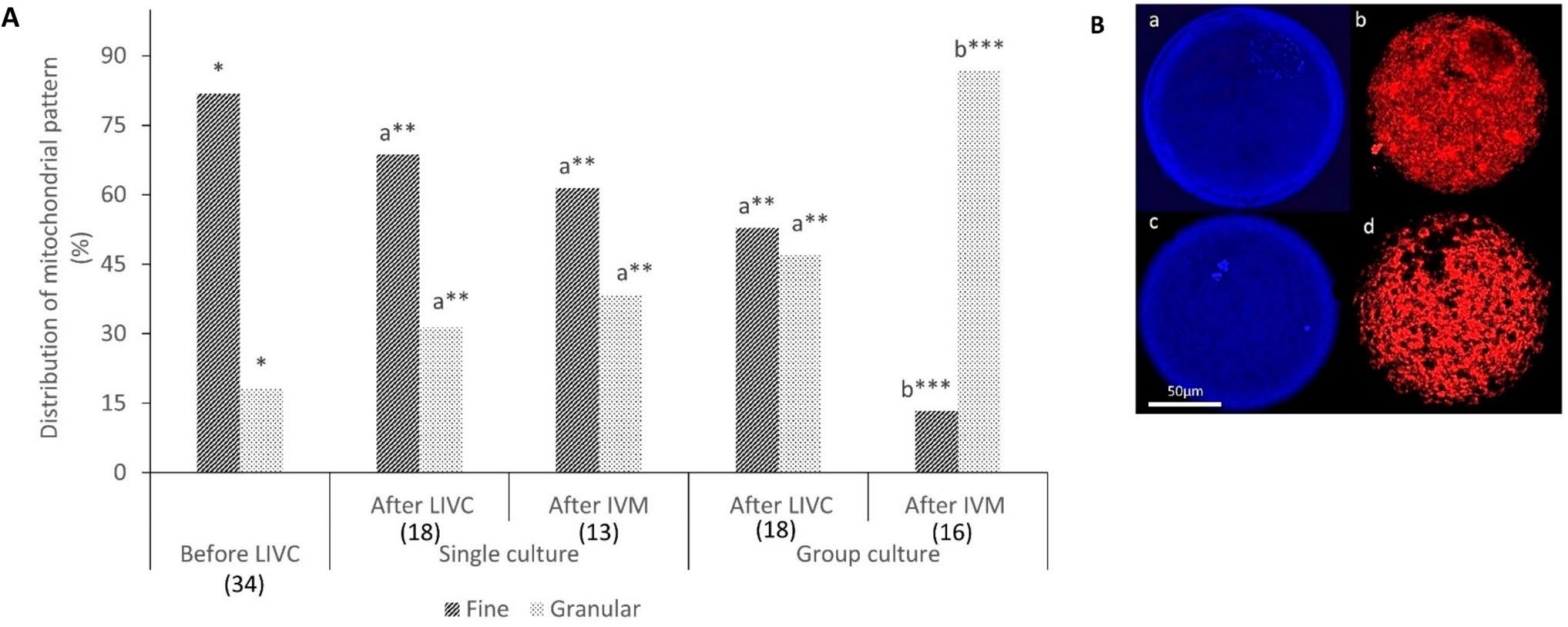
Mitochondrial distribution patterns in freshly isolated oocytes following LIVC and IVM. **A** The bar graph shows the percentage of oocytes exhibiting different mitochondrial distribution patterns: “fine” and “granular”. The data are presented for freshly isolated oocytes from EAFs and following procedures of LIVC and IVM. **B** Representative confocal images of mitochondrial distribution patterns in oocytes, stained with MitoTracker Red (b and d) and Hoechst dye (a and c), to illustrate “fine” (b) and “granular” (d) patterns at the GV (a) and MII (c) stages. Different letters indicate significant differences within the group. Asterisks (*) indicate significant differences between groups (*p* < 0.05)

In contrast to mitochondrial activity, the analysis of mitochondrial distribution revealed significant differences between the groups, with only two classes of distribution observed: “fine” and “granular” (Fig. 8A and B). Most of the freshly isolated oocytes initially exhibited a “fine” distribution pattern (81.8%). After LIVC, a significant transition from a fine to a granular distribution was observed, particularly in the group culture (*p* < 0.001). These changes in mitochondrial distribution continued following IVM, with a more pronounced shift toward the “granular” pattern in the group culture compared to the single culture group (Fig. 8, *p* < 0.05).

### Effect of LIVC on meiotic competence

**Fig. 9:**
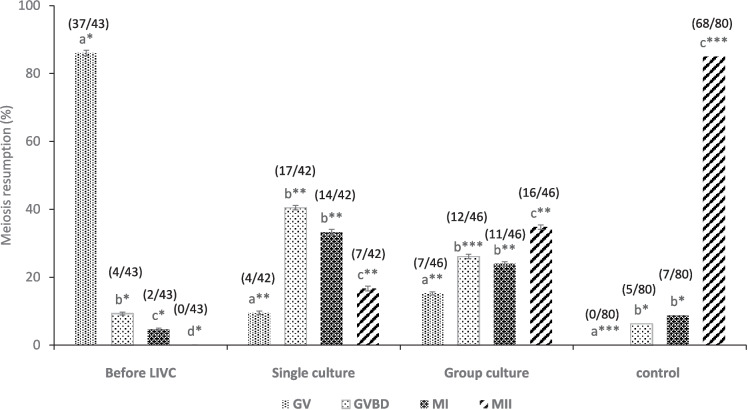
Meiotic progression of oocytes after IVM. The graph illustrates the distribution of oocytes at various meiotic stages—germinal vesicle (GV), germinal vesicle breakdown (GVBD), metaphase I (MI), and metaphase II (MII)—following IVM. The data compares oocytes freshly isolated from AFs, EAFs, and after LIVC in both single and group cultures. Numbers in parentheses indicate the evaluated COCs. Different letters indicate significant differences within the group. Asterisks (*) indicate significant differences between groups (*p* < 0.05)

Before LIVC, the majority of COCs that underwent in vitro maturation remained at the GV (37/43) or GVBD (4/43) stages (Fig. 9). After LIVC, enhanced meiotic resumption, progressing to metaphase I (MI) and metaphase II (MII), was observed in both single (21/42) and group cultures (27/46), indicating successful meiotic resumption (*p* < 0.05). Notably, a higher percentage of oocytes reached the MII stage in group culture compared to single culture (34.78% vs. 16.67%, *p* < 0.05). This outcome was accompanied by a reduction in the proportion of oocytes detained at the GV stage, suggesting that LIVC may support the acquisition of oocyte meiotic competence under group culture conditions. However, despite the promising rate of meiotic resumption in both groups, these rates remained substantially lower than those of oocytes from antral follicles (Fig. 9; *p* < 0.01).

## Discussion

The present study demonstrates that group culture significantly influences the outcomes of LIVC of COCs derived from sheep EAFs. This effect is facilitated by enhanced cellular communication, as evidenced by sustained gap junction functionality and improved developmental potential, indicated by greater meiotic competence and a favorable redistribution of cellular organelles.

Although no significant differences in morphological quality and viability were observed between the two groups, COCs in the group culture displayed a distinct cluster-like structure with tightly packed granulosa cells. This clustering may promote mutual protection and support through close cellular interactions, which help to preserve COC integrity. While this setup does not fully replicate the follicular structure found in vivo, clustering COCs in vitro may still simulate aspects of the ovarian microenvironment, as follicles in the ovary also grow close to each other [43, 44]. Supporting this concept, Hornick et al. (2013) demonstrated that group culture of preantral follicles in mice helps maintain follicular structure and promotes survival in a number-dependent manner. Similarly, co-culture systems have been shown to enhance the developmental potential of denuded oocytes by providing a supportive cellular environment [45, 46].

These findings suggest that similar mechanisms may operate within group cultures of COCs, emphasizing the role of mutual support and an enhanced microenvironment in facilitating oocyte growth and metabolism, further supported by ultrastructural analyses and observations of mitochondrial distribution. This stage is marked by the initiation of perivitelline space formation, resulting in shorter and bent microvilli and liberation from the ZP [47], which aligns with our results. Additionally, the ZP exhibited a thinner, continuous structure, and the mitochondria transitioned into a hooded shape at the oocyte periphery, a change more prevalent during the later stages of oocyte growth in sheep and cows [13, 47]. This shape alteration may indicate shifts in the energetic state of the mitochondria [13, 48].

Regarding mitochondrial distribution, oocytes from the group culture exhibited more peripherally located mitochondria, consistent with the distribution observed in immature oocytes from antral follicles in sheep [48]. Regarding vesicles, group-cultured oocytes exhibited a lower number of them. Typically, ovine oocytes contain a large number of vesicles, which harbor a mixture of metabolites essential for oocyte maturation and development [13, 49]. Therefore, the reduced number of vesicles in the group-cultured oocytes might be attributed to increased vesicle utilization, potentially enhancing oocyte quality and competency [13].

Evaluation of GJCs in group culture demonstrated a higher prevalence of functional gap junctions (open and partially open) compared to single culture (*p* < 0.05), supporting the notion that group culture may play a crucial role in maintaining oocyte-cumulus cell communication through the upregulation of key transcription factors [50] and paracrine signaling from surrounding granulosa cells [7, 15, 21, 50, 51]. This may facilitate the transfer of soluble factors from cumulus cells to the oocyte, supporting oocyte development and contributing to the improved meiotic progression observed in group cultures [45]. In contrast, single-cultured COCs may not benefit from the mutual support provided by proximity to other COCs.

Regarding the oocyte diameter, which is related to the increased amount of organelles and the accumulation of metabolites [38], both experimental groups exhibited an increase in oocyte diameter but did not reach the dimensions of fully grown oocytes isolated from antral follicles (133.6 ± 4.5 µm, data not shown). Sheep oocytes typically achieve full developmental competence at approximately 120.0 ± 3.0 µm [52], suggesting that reaching this threshold size (≥ 120.0 µm) may be more critical than achieving the maximum size observed in antral follicles. Since our LIVC system supports an oocyte growth rate of 1.16 to 1.38 µm/day, extending the culture period may facilitate oocytes reaching the threshold diameter of 120.0 µm. Alternatively, selecting larger oocytes that are more likely to attain this threshold within 5 days of LIVC could be an effective strategy to enhance outcomes.

Although the oocytes did not reach the expected size of fully grown oocytes, significant chromatin changes were observed in both groups following LIVC. This was evidenced by the transition from NSN to SN configuration, indicating the efficiency of LIVC in chromatin condensation, a critical process in oocyte differentiation. This transition is also associated with the progressive silencing of transcriptional activity, a hallmark of the oocyte’s advancement toward the fully grown stage [18, 26], which aligns with our results.

The observed enhancement in meiotic resumption (MI + MII) following IVM, particularly noted in the group culture system, underscores the crucial support provided by granulosa cells. These cells release paracrine signals into the culture environment, which are pivotal in activating follicles and indirectly promoting the progression of meiosis [53]. This phenomenon aligns with findings by Jones et al. (2021), who reported enhanced meiotic resumption in group-cultured murine preantral follicles [54]. They attributed this improvement to the upregulation of pathways, including calcineurin, Wnt, prolactin, and angiogenic signaling. These insights highlight the significance of the biochemical environment in group culture settings, where the collective influence of multiple signaling pathways fosters a more conducive milieu for oocyte maturation.

To assess cytoplasmic maturation, we also explored mitochondrial activity and distribution patterns, which are critical during oocyte development [55]. Interestingly, mitochondrial activity in oocytes remained unchanged post-LIVC in both experimental groups compared to freshly isolated oocytes. This stability suggests that the LIVC system effectively preserves mitochondrial function, a vital aspect of maintaining oocyte viability and competence.

Despite established associations between higher mitochondrial activity and both meiosis progression and developmental potential [37], such trends were not evident during LIVC in our experiments. This suggests that while LIVC maintains mitochondrial function, it does not necessarily enhance it. However, after undergoing IVM, we noted a significant increase in mitochondrial activity, indicating that the activation of mitochondrial function might be more effectively triggered during the maturation phase rather than during the culture phase. This increase occurred irrespective of single- or group-culture conditions, suggesting that the enhancement of mitochondrial activity is a general response to IVM rather than being influenced by specific culture environments (i.e., LIVC vs. IVM).

During oocyte maturation in pigs and cattle, an increase in fluorescence intensity of labeled mitochondria correlates with the rising demand for ATP necessary for activation and subsequent embryo development [13, 56]. However, similar studies on sheep have not demonstrated significant changes in mitochondrial activity from GV to MII transition [55], highlighting species-specific variations in mitochondrial behavior during oocyte maturation. These contradictory findings across different species may stem from various factors, including differences in culture media, the specific conditions under which the cultures are maintained (such as variations in oxygen tension), or even the types of mitochondrial stains used, which can affect the visibility and apparent activity of mitochondria [57]. Such variables underscore the complex interplay of environmental and biological factors influencing mitochondrial dynamics during oocyte maturation.

Regarding the mitochondrial distribution pattern, both freshly isolated and single-cultured oocytes post-LIVC and IVM predominantly exhibited a fine mitochondrial distribution pattern. It has been reported that a fine distribution pattern is correlated with poor developmental oocyte competence. In contrast, a granular and/or clustered distribution pattern is related to a well-organized microtubule network involved in the translocation of mitochondria [30, 58]. This translocation is crucial as it is mediated by microtubule networks and their associated motor proteins [59]. Therefore, the observed granular pattern in group culture, along with the higher maturation rate after IVM, indicates the greater developmental competence of group-cultured COCs to complete the first meiotic division.

In contrast, the association between fine mitochondrial patterns and poorer outcomes highlights the need for further investigation into how these patterns influence the acquisition of oocyte developmental competence. Additionally, it remains to be explored whether adjustments in culture conditions could promote more favorable mitochondrial distribution patterns. Based on these observations, mitochondrial distribution patterns are a more reliable indicator of cytoplasmic maturation than mitochondrial activity alone, warranting further research into optimizing culture environments to enhance oocyte quality.

Additionally, our findings indicated no significant changes in the intracellular levels of ROS before and after LIVC and IVM in both culture conditions, suggesting that the LIVC system may exert a protective effect, maintaining a balanced redox state within the oocytes. This effect could be attributed to the presence of cysteamine in the medium, which increases cysteine uptake and promotes glutathione synthesis, thereby reducing oxidative stress [60, 61]. However, the potential indirect roles of components like GlutaMAX™ and sodium pyruvate in reducing oxidative stress by supporting cellular metabolism should not be overlooked.

It is essential to acknowledge the limitations of our study, including the use of abattoir-derived ovaries, which resulted in variability in follicle quality due to unknown animal age and reproductive cycle stage. Despite advancements in 3D culture techniques and the introduction of culture medium supplements, mimicking the natural environment for in vitro follicle growth remains challenging. Further research is needed to develop culture systems that better support morphological integrity and metabolic requirements. Additionally, maintaining COCs’ proximity within flat-bottomed wells presented a technical challenge, which could be mitigated using v-shaped wells to reduce movement and help COCs remain closer together.

In conclusion, this study demonstrates that although LIVC supports oocyte growth in both single and group cultures, group culture offers significant advantages. Specifically, group culture promotes granulosa cell clustering, sustains (promotes) gap junction functionality, optimizes mitochondrial distribution, and induces gradual chromatin configuration changes, all of which contribute to acquiring meiotic and developmental competencies.

These findings underscore the potential of group culture in designing more effective in vitro culture systems for EAFs, potentially enhancing oocyte growth and maturation outcomes. Further studies should identify the molecules driving the positive effects in group culture, refine culture conditions, and assess mitochondrial distribution patterns as oocyte competence markers. Progress in this area could greatly expand the use of EAF pools, thereby improving ovarian reserve exploitation for fertility preservation and the treatment of various reproductive disorders. Moreover, this model system provides a valuable platform for investigating the interplay and paracrine signaling among COCs. These findings suggest that optimized group culture conditions may enhance outcomes in IVM systems, offering valuable insights for reproductive science and biotechnology applications.

## Supplementary Information

Below is the link to the electronic supplementary material.

Supplementary file1 (JPG 1388 KB)

## Data Availability

The data supporting the findings of this study are available upon reasonable request from the corresponding author.
