# Supplementary figures and images for: The effect of single versus group culture on cumulus-oocyte complexes from early antral follicles
Source: J Assist Reprod Genet. 2025 Jan 28;42(3):961–76. doi: 10.1007/s10815-025-03404-w (PMC11950561; doi:10.1007/s10815-025-03404-w)

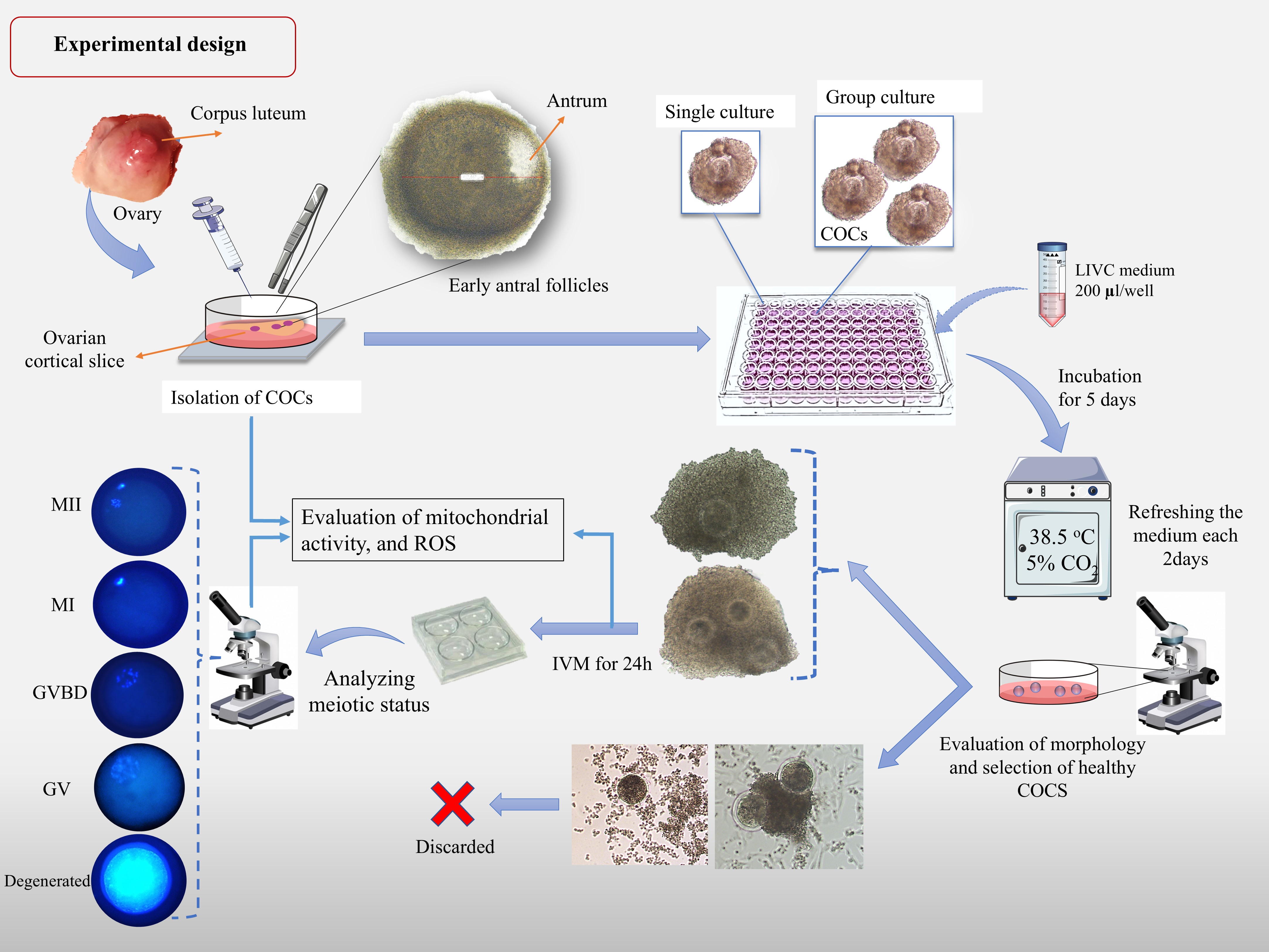

Supplement: Supplementary file 1 — Supplementary file1 (JPG 1388 KB) [file 10815_2025_3404_MOESM1_ESM.jpg]
